# Supplementary material for: Human skeletal muscle organoids model fetal myogenesis and sustain uncommitted PAX7 myogenic progenitors
Source: eLife. 2023 Nov 14;12:RP87081. doi: 10.7554/eLife.87081 (PMC10645425; doi:10.7554/eLife.87081)
Supplement: Supplementary file 1. — (a) qPCR primer pairs applied to detect relative expression of key markers during skeletal muscle organoid development. (b) qPCR primer pairs applied for diffusion map analysis of early skeletal muscle organoid development. [file elife-87081-supp1.docx]

| **Gene** | **Forward Primer Sequence** | **Reverse Primer Sequence** |
| --- | --- | --- |
| **OCT4** | AGTGAGAGGCAACCTGGAGA | ACACTCGGACCACATCCTTC |
| **SOX2** | GCCCTGCAGTACAACTCCAT | GACTTGACCACCGAACCCAT |
| **NANOG** | CTGAGATGCCTCACACGGAG | GTGGGTTGTTTGCCTTTGGG |
| **PAX6** | GTGAGAGTGGACAGACATCCG | CTGTTCTGCATGCTGGCTCT |
| **BRACHYURY** | TTCATAGCGGTGACTGCTTATCA | CACCCCCATTGGGAGTACC |
| **MESOGENIN** | CAGGATGAGGACCTTGGCAG | GGATCTTGGTGAGAGGCTGG |
| **TBX6** | CCCTACTCGGCTGCATTTCT | GAGCCCACATCCAGATAGCC |
| **HES7** | GGAACCCGAAGCTGGAGAAA | CGGAAACCGGACAAGTAGCA |
| **UNCX** | GGAGAAGGCGTTCAACGAGA | GGAACCAGACCTGAACTCGG |
| **TBX18** | CCACCCCGTGTGTACATTCA | TGGCCTTGGTCATCCAGTTC |
| **MEOX2** | CTCTGCAAACCAACTGGCAC | AAGAGTTGGAGCACAGGACG |
| **PAX3** | AGACTGGCTCCATACGTCCT | CATGCCCGGGTTCTCTCTTT |
| **PAX7** | AACCACATCCGCCACAAGAT | CTCCTGGTAGCGGCAAAGAA |
| **EN1** | CAGGAACTCAGCCTCAACGA | ACTCGCTCTCGTCTTTGTCC |
| **SIM1** | GAGTGGTGTTCCCAGAAGGG | ATCCAGGGTCTGGAGCAGAT |
| **LBX1** | GCGGAGAAGTTACTCGCTGT | CCTTAAACGTCTTGCTGGCG |
| **TFAP2A** | CCAAGTCCAACAGCAATGCC | CGACCCGGAACTGAACAGAA |
| **SOX10** | CCATCCAGGCCCACTACAAG | GCTCTGGCCTGAGGGGT |
| **RPS16**  **(Housekeeping)** | GCTATCCGTCAGTCCATCTCCAA | CCTTCTTGGAAGCCTCATCCAC |

**Supplementary file 1a**

| **Gene** | **Forward Primer Sequence** | **Reverse Primer Sequence** |
| --- | --- | --- |
| **OCT4** | GTGTTCAGCCAAAAGACCATCT | GGCCTGCATGAGGGTTTCT |
| **NANOG** | TTTGTGGGCCTGAAGAAAACT | AGGGCTGTCCTGAATAAGCAG |
| **EOMES** | TCCATCTCCCACGGATTCTC | TTCGCTCTGTTGGGGTGAAA |
| **SOX17** | GTGGACCGCACGGAATTTG | GGAGATTCACACCGGAGTCA |
| **PAX6** | AACGATAACATACCAAGCGTGT | GGTCTGCCCGTTCAACATC |
| **SOX2** | TGGACAGTTACGCGCACAT | CGAGTAGGACATGCTGTAGGT |
| **MSX1** | ACACAAGACGAACCGTAAGCC | CACATGGGCCGTGTAGAGTC |
| **IRX3** | CCTGTCCAACGTGCTCTCG | GGCCATACGGGTAGAAGGC |
| **WNT3A** | GTGTTCCACTGGTGCTGCTA | CCCTGCCTTCAGGTAGGAGT |
| **FGF8** | GACCCCTTCGCAAAGCTCAT | CCGTTGCTCTTGGCGATCA |
| **BRACHYURY** | TATGAGCCTCGAATCCACATAGT | CCTCGTTCTGATAAGCAGTCAC |
| **MSGN** | GGCCTGGTAGAGGTGGACTA | ACAGGTGGCAGGTAATTCCG |
| **TBX6** | CATCCACGAGAATTGTACCCG | AGCAATCCAGTTTAGGGGTGT |
| **FOXC1** | GGCGAGCAGAGCTACTACC | TGCGAGTACACGCTCATGG |
| **RALDH2** | GCCAGGAGCTGGTACACTAC | AGAAGCGGTGAACTGCACAT |
| **RIPPLY2** | GCTTTACCAATTCAGGCACCC | CACAGGTCAGATCCTCAATTTCA |
| **FOXC2** | CCTCCTGGTATCTCAACCACA | GAGGGTCGAGTTCTCAATCCC |
| **TCF15** | CCTCCTTCTCCAGGGTCCAG | CAGCTGCTTGAAGGTGAGGG |
| **MEOX1** | TCGGCTCCGCAGATATGAGA | CCCTTCACACGCTTCCACTT |
| **PAX3** | AGACTGGCTCCATACGTCCT | CATGCCCGGGTTCTCTCTTT |
| **PAX1** | AGACGTATGGCGAGGTGAAC | CCGACTGATGTCACAGGGTC |
| **PAX9** | CCCCAATTCCCAGGTCTCAC | AGTCCGTACAGCCAGCTTTC |
| **MAFB** | TCAAGTTCGACGTGAAGAAGG | GTTCATCTGCTGGTAGTTGCT |
| **ZIC3** | GGCGCTCAGTTTCCTAACTAC | CTGCCGCATATAACGGAAGAA |
| **GDF7** | TGATGTCGCTTTACCGGAGC | CTGCCGATTCGTCTTGGGT |
| **TFAP2A** | AGGTCAATCTCCCTACACGAG | GGAGTAAGGATCTTGCGACTGG |
| **ETS1** | GATAGTTGTGATCGCCTCACCC | GTCCTCTGAGTCGAAGCTGTC |
| **PAK3** | CCAGGCTTCGCTCTATCTTCC | TCAAACCCCACATGAATCGTATG |
| **FOXD3** | GCAACTACTGGACCCTGGAC | CTGTAAGCGCCGAAGCTCT |
| **OSR1** | CAAGCCGCGCTTTGATTTTG | TCCGCTCATGGATAAGTAGGTT |
| **PAX2** | TGTCAGCAAAATCCTGGGCAG | GTCGGGTTCTGTCGTTTGTATT |
| **LHX1** | CATGCGCGTCATTCAGGTCT | GAGAAGGGACCATTGGGGAT |
| **RPS16**  **(Housekeeping)** | GCTATCCGTCAGTCCATCTCCAA | CCTTCTTGGAAGCCTCATCCAC |
| **RPL37A**  **(Housekeeping)** | GTGGTTCCTGCATGAAGACAGTG | TTCTGATGGCGGACTTTACCG |

**Supplementary file 1b**
