# Supplementary material for: Human skeletal muscle organoids model fetal myogenesis and sustain uncommitted PAX7 myogenic progenitors
Source: eLife. 2023 Nov 14;12:RP87081. doi: 10.7554/eLife.87081 (PMC10645425; doi:10.7554/eLife.87081)
Supplement: MDAR checklist [file elife-87081-mdarchecklist1.docx]

**Materials Design Analysis Reporting (MDAR)**

**Checklist for Authors**

The [MDAR framework](https://osf.io/xfpn4/) establishes a minimum set of requirements in transparent reporting mainly applicable to studies in the life sciences.

*eLife* asks authors to **provide detailed information within their article** to facilitate the interpretation and replication of their work. Authors can also upload supporting materials to comply with relevant reporting guidelines for health-related research (see [EQUATOR Network](http://www.equator-network.org/%20)), life science research (see the [BioSharing Information Resource](http://biosharing.org/)), or animal research (see the [ARRIVE Guidelines](http://www.plosbiology.org/article/info:doi/10.1371/journal.pbio.1000412) and the [STRANGE Framework](https://doi.org/10.1038/d41586-020-01751-5); for details, see *eLife*’s [Journal Policies](https://reviewer.elifesciences.org/author-guide/journal-policies)). Where applicable, authors should refer to any relevant reporting standards materials in this form.

For all that apply, please note **where in the article** the information is provided. Please note that we also collect information about data availability and ethics in the submission form.

**Materials:**

| **Newly created materials** | **Indicate where provided: section/figure legend** | **N/A** |
| --- | --- | --- |
|  |  | N/A |
|  |  |  |
| **Antibodies** | **Indicate where provided: section/figure legend** | **N/A** |
| *Primary Antibodies*: anti-Brachyury (R&DSystems, 1:250), anti-TBX6(Abcam, 1:200), anti-PAX3 (DHSB, 1:250), anti- PAX7(DHSB, 1:250), anti-SOX10 (R&DSystems,1:125), anti-KI67 (ThermoFisher Scientific, clone SolA15, 1:100), anti- TITIN (DHSB, 9D-10, 1:300), anti-MyHC (DHSB, MF20, 1:300), anti-MYOD1 (Santa Cruz Biotechnologies, clone 5.8A, 1:200), anti-PRDM16 (Abcam, ab106410, 1:200), anti-TFAP2A (DHSB, 3B5, 1:100), anti-Dystrophin (Novocastra/Leica Biosystems, clone DY4/6D3, 1:200), anti-Laminin (SigmaAldrich, 1:200), anti-FastMyHC (SigmaAldrich, clone MY-32, 1:300), anti-M-Cadherin (Cell Signaling Technology, 1:200) anti-SOX2 (ThermoFisher Scientific, clone Btjce, 1:100), anti-CD44 (eBioscience, clone IM7, 1:100), anti-Fibrillin1 (Invitrogen, clone 11C1.3, 1:100). *Secondary antibodies*: Alexa Fluor® 647 AffiniPure Fab Fragment Goat Anti-Mouse IgM, μ Chain Specific (Jackson Immunoresearch Laboratories, 1:100), Rhodamine RedTM-X (RRX) AffiniPure Goat Anti-Mouse IgG, Fcγ Subclass 1 Specific (Jackson Immunoresearch Laboratories,1:100), Alexa Fluor® 488 AffiniPure Goat Anti-Mouse IgG, Fcγ subclass 2a specific (Jackson Immunoresearch Laboratories,1:100), Alexa Fluor 488, Goat anti-Rat IgG (H+L) Cross- Adsorbed Secondary Antibody, (ThermoFisher Scientific, 1:500), Alexa Fluor 488, Donkey anti-Mouse IgG (H+L) Cross-Adsorbed Secondary Antibody, (ThermoFisher Scientific, 1:500), Alexa Fluor 647, Donkey anti-Goat IgG (H+L) Cross-Adsorbed Secondary Antibody, (ThermoFisher Scientific, 1:500), Alexa Fluor 488, Donkey anti-Goat IgG (H+L) Cross-Adsorbed Secondary Antibody, (ThermoFisher Scientific, 1:500), Alexa Fluor 568, Donkey anti-Rabbit IgG (H+L) Cross-Adsorbed Secondary Antibody, (ThermoFisher Scientific, 1:500). | Figures  1 C,D,G,H  2 C,D,E  3 B,C,H,J  4 B,C,I,J  1S1 C,D,E  2S1 D  3S1 D,E  3S2 F  3S3 C,D  4S1 C  4S3 C |  |
|  |  |  |
| **DNA and RNA sequences** | **Indicate where provided: section/figure legend** | **N/A** |
| qPCR primer pairs to measure relative gene expression  qPCR primer pairs for diffusion map analysis | Table S1  Table S2 |  |
|  |  |  |
| **Cell materials** | **Indicate where provided: section/figure legend** | **N/A** |
| Cord Blood iPSC, Max Planck Institute Münster, (Dorn et al., 2015)  DMD_iPS1, BMD_iPS1, Boston Children’s Hospital Stem Cell Core (Park et al., 2008)  DMD_iPSCORE_65_1, WiCell cat.no. WB60393 (Panopoulos et al., 2017)  LGMD2A + isogenic iPSC, Ruhr University Bochum (Mavrommatis et al., 2020) | all study |  |
|  |  |  |
|  |  |  |
| **Experimental animals** | **Indicate where provided: section/figure legend** | **N/A** |
| 2-3 months old male HsdCpb:NMRI-Foxn1nu mice  Harlan Laboratories B.V., PO Box 553, 5800 AN Venray oder JANVIER SAS, CS 4105, LE GENEST ST ISLE F-53941 St Berthevin Cedex | Figure 4 I,J |  |
|  |  |  |
|  |  |  |
| **Plants and microbes** | **Indicate where provided: section/figure legend** | **N/A** |
|  |  | N/A |
|  |  |  |
|  |  |  |
| **Human research participants** | **Indicate where provided: section/figure legend) or state if these demographics were not collected** | **N/A** |
|  |  | N/A |

**Design:**

| **Study protocol** | **Indicate where provided: section/figure legend** | **N/A** |
| --- | --- | --- |
|  |  | N/A |
|  |  |  |
| **Laboratory protocol** | **Indicate where provided: section/figure legend** | **N/A** |
|  |  | N/A |
|  |  |  |
| **Experimental study design (statistics details) *** | | |
| **For in vivo studies: State whether and how the following have been done** | **Indicate where provided: section/figure legend. If it could have been done, but was not, write “not done”** | **N/A** |
| Sample size determination |  | N/A |
| Randomisation |  | N/A |
| Blinding |  | N/A |
| Inclusion/exclusion criteria |  | N/A |
|  |  |  |
| **Sample definition and in-laboratory replication** | **Indicate where provided: section/figure legend** | **N/A** |
| The organoid approach was evaluated with six hiPSCs lines with independent genetic backgrounds, with more than five independent derivations per line, especially for the control line (CB CD34+) more than 20 derivation, obtaining always similar results. Our reported protocol is based on about 45 independent differentiation inductions. | Figure 1-4 |  |
| Each hiPSC line or independent organoid derivation from the same line in different passages were considered as biological replicates, while replicates from same derivations as technical ones. | Figure 1-4 |  |
|  |  |  |
| **Ethics** | **Indicate where provided: section/submission form** | **N/A** |
| Studies involving human participants: State details of authority granting ethics approval (IRB or equivalent committee(s), provide reference number for approval. | M&M: No experiments on human participants were performed. The use of reprogrammed human iPSC lines for research was performed after ethical approval from the ethics commission of the Ruhr-University Bochum, Medical Faculty (15-5401, 08/2015). | N/A |
| Studies involving experimental animals: State details of authority granting ethics approval (IRB or equivalent committee(s), provide reference number for approval. | M&M: All animal experiments were approved by the local authorities (81-02.04.2020.A476, Ruhr University Bochum) and performed in accordance with the guidelines for Ethical Conduct in the Care and Use of Animals. |  |
| Studies involving specimen and field samples: State if relevant permits obtained, provide details of authority approving study; if none were required, explain why. |  | N/A |
|  |  |  |
| **Dual Use Research of Concern (DURC)** | **Indicate where provided: section/submission form** | **N/A** |
| If study is subject to dual use research of concern regulations, state the authority granting approval and reference number for the regulatory approval. |  | N/A |

**Analysis:**

| **Attrition** | **Indicate where provided: section/figure legend** | **N/A** |
| --- | --- | --- |
| Describe whether exclusion criteria were pre-established. Report if sample or data points were omitted from analysis. If yes, report if this was due to attrition or intentional exclusion and provide justification. |  | N/A |
|  |  |  |
| **Statistics** | **Indicate where provided: section/figure legend** | **N/A** |
| All statistical analysis was conducted using GraphPad Prism6 software. For qPCR analysis one-way ANOVA with Tukey ́s multiple comparisons test for each marker was performed. For the FACS intracellular staining quantification, one-way ANOVA with Sidak ́s multiple comparisons test between the different time points was performed. Significance asterisks represent *P < 0.05, **P < 0.01, ***P < 0.001, ****P < 0.0001, ns: not significant | Figure 2F, 1S1A,  1S1B, 2S1D, 3S2E |  |
|  |  |  |
| **Data availability** | **Indicate where provided: section/submission form** | **N/A** |
| For newly created and reused datasets, the manuscript includes a data availability statement that provides details for access (or notes restrictions on access). |  |  |
| When newly created datasets are publicly available, provide accession number in repository OR DOI and licensing details where available. | RNA sequencing datasets produced in this study are deposited in the Gene Expression Omnibus (GEO) under accession code GSE147514. Detailed scripts and parameters used for the study are available from the authors upon reasonable request.  To review GEO accession GSE147514: Go to https://www.ncbi.nlm.nih.gov/geo/query/acc.cgi?acc=GSE147514 |  |
| If reused data is publicly available provide accession number in repository OR DOI, OR URL, OR citation. | Embryonic and fetal (weeks 5 to 18) and adult satellite cell (years 7,11,34,42) scRNAseq data are from Xi et al., 2020 (GSE147457), adult satellite cell (year 25) scRNAseq data are from Rubenstein et al., 2020 (GSE130646). |  |
|  |  |  |
| **Code availability** | **Indicate where provided: section/figure legend** | **N/A** |
| For any computer code/software/mathematical algorithms essential for replicating the main findings of the study, whether newly generated or re-used, the manuscript includes a data availability statement that provides details for access or notes restrictions. |  | N/A |
| Where newly generated code is publicly available, provide accession number in repository, OR DOI OR URL and licensing details where available. State any restrictions on code availability or accessibility. |  | N/A |
| If reused code is publicly available provide accession number in repository OR DOI OR URL, OR citation. |  | N/A |

**Reporting:**

The MDAR framework recommends adoption of discipline-specific guidelines, established and endorsed through community initiatives.

| **Adherence to community standards** | **Indicate where provided: section/figure legend** | **N/A** |
| --- | --- | --- |
| State if relevant guidelines (e.g., ICMJE, MIBBI, ARRIVE, STRANGE) have been followed, and whether a checklist (e.g., CONSORT, PRISMA, ARRIVE) is provided with the manuscript. |  | N/A |

* We provide the following guidance regarding transparent reporting and statistics; we also refer authors to [Ten common statistical mistakes to watch out for when writing or reviewing a manuscript](https://doi.org/10.7554/eLife.48175).

**Sample-size estimation**

- You should state whether an appropriate sample size was computed when the study was being designed
- You should state the statistical method of sample size computation and any required assumptions
- If no explicit power analysis was used, you should describe how you decided what sample (replicate) size (number) to use

**Replicates**

- You should report how often each experiment was performed
- You should include a definition of biological versus technical replication
- The data obtained should be provided and sufficient information should be provided to indicate the number of independent biological and/or technical replicates
- If you encountered any outliers, you should describe how these were handled
- Criteria for exclusion/inclusion of data should be clearly stated
- High-throughput sequence data should be uploaded before submission, with a private link for reviewers provided (these are available from both GEO and ArrayExpress)

**Statistical reporting**

- Statistical analysis methods should be described and justified
- Raw data should be presented in figures whenever informative to do so (typically when N per group is less than 10)
- For each experiment, you should identify the statistical tests used, exact values of N, definitions of center, methods of multiple test correction, and dispersion and precision measures (e.g., mean, median, SD, SEM, confidence intervals; and, for the major substantive results, a measure of effect size (e.g., Pearson's r, Cohen's d)
- Report exact p-values wherever possible alongside the summary statistics and 95% confidence intervals. These should be reported for all key questions and not only when the p-value is less than 0.05.

**Group allocation**

- Indicate how samples were allocated into experimental groups (in the case of clinical studies, please specify allocation to treatment method); if randomization was used, please also state if restricted randomization was applied
- Indicate if masking was used during group allocation, data collection and/or data analysis
